# Environmental Chemical Exposomics and Metabolomics in Toxicology: Recent Advances

**DOI:** 10.3390/toxics14090803

**Published:** 2026-09-10

**Authors:** Minjian Chen

**Affiliations:** 1 Department of Occupational Medicine and Environmental Health, School of Public Health, Key Laboratory of Public Health Safety and Emergency Prevention and Control Technology of Higher Education Institutions in Jiangsu Province, Nanjing Medical University, Nanjing 211166, China; minjianchen@njmu.edu.cn; 2 State Key Laboratory of Reproductive Medicine and Offspring Health, Center for Global Health, School of Public Health, Nanjing Medical University, Nanjing 211166, China; 3 Key Laboratory of Modern Toxicology of Ministry of Education, School of Public Health, Nanjing Medical University, Nanjing 211166, China

## 1. Introduction

This editorial introduces the Special Issue titled “State-of-the-Art Environmental Chemical Exposomics and Metabolomics—2nd Edition.” Due to the complexity and diversity of human exposure to environmental factors, increasing attention has been devoted to understanding exposure–health relationships from a more comprehensive perspective. Chemical exposomics provides an important framework for characterizing complex chemical exposures and identifying their potential health risks [1,2,3]. Metabolomics systematically profiles endogenous small-molecule substrates, intermediates, and products of cellular metabolism, which are closely related to phenotypes and provide important information on physiological and pathological processes [4]. Integrating environmental chemical exposomics with metabolomics and complementary molecular information can therefore facilitate the identification of key environmental chemicals and their associated metabolic signatures and help elucidate mechanisms linking chemical exposure to adverse health outcomes [5,6,7].

This Special Issue contains eight research papers. Building on Volume I [8], this second edition further covers external and internal chemical exposure characterization, metabolomics and its integration with other omics, and experimental and computational investigations of metabolism-related pathways and molecular targets.

## 2. An Overview of Published Articles

Characterizing both external and internal exposure is essential for comprehensively evaluating the chemical exposome. Environmental monitoring using broad chemical detection can reveal complex external exposure sources, whereas human biomonitoring provides information on chemicals that have entered the body and can be linked more directly to health outcomes. Walker-Franklin et al. (contribution 1) investigated chemical contamination in the French Broad River following Hurricane Helene using targeted PFAS analysis combined with liquid chromatography–high-resolution mass spectrometry-based non-targeted analysis and suspect screening. Eleven PFASs were detected, while non-targeted analysis and suspect screening identified 468 compounds, including 96 compounds with high-confidence structural annotations. Cheminformatics-based hazard screening further indicated substantial aquatic and human health hazard potential among the detected contaminants, while PFOA and PFOS at the most contaminated site exceeded the U.S. EPA drinking-water maximum contaminant levels. The study demonstrated that extreme flooding can mobilize diverse anthropogenic contaminants and highlighted the value of combining targeted and non-targeted approaches for characterizing complex external chemical exposures. Li et al. (contribution 2) focused on internal exposure by simultaneously examining 33 personal care and consumer product chemicals, including PFASs, phthalates, phenols, and parabens, in 684 women from the National Health and Nutrition Examination Survey. Elevated PFAS exposure, particularly PFHxS and n-PFOA, was associated with long-term amenorrhea. Serum globulin partially mediated these associations, and further target analysis suggested STAT3 as a potential molecular target. Notably, PFOA emerged as a chemical of concern in both studies. Together, these studies demonstrate how chemical exposome assessment can extend from complex external environmental contamination to internal mixed exposure, thereby facilitating the identification of priority environmental chemicals and their potential health effects.

Metabolomics provides molecular information that is close to the phenotype and is therefore particularly valuable for linking environmental chemical exposures to adverse health outcomes in both human and animal studies. Zhao et al. (contribution 3) provided a direct example of integrating internal chemical exposure with metabolomics. Whole-blood barium concentrations were measured in 183 pregnant women, and untargeted metabolomics of decidual tissues detected 523 metabolites. Nineteen metabolites and five metabolic pathways were associated with both barium exposure and miscarriage, with glycerophospholipid metabolism being particularly prominent. Five metabolites were further identified as potential mediators of the association between barium and miscarriage, and a metabolite-based XGBoost model achieved an AUC of 0.90 for miscarriage prediction. The study established a direct link among environmental exposure, endogenous metabolic alterations, and adverse reproductive outcomes. Bai et al. (contribution 4) investigated the intergenerational effects of perinatal exposure to bisphenol AF (BPAF), an increasingly used BPA substitute. BPAF exposure induced persistent metabolic abnormalities in offspring, including glucose intolerance and hepatic steatosis. Integrated epigenomic, transcriptomic, and metabolomic analyses showed genome-wide alterations in H3K27ac-marked regulatory elements, suppression of immune-related responses, activation of sterol biosynthesis, and perturbation of the hepatic metabolome, including depleted pantothenate and increased pro-inflammatory eicosanoids. The study demonstrates how environmental exposure can induce persistent metabolic changes through coordinated regulation across the epigenome, transcriptome, and metabolome.

The investigation of metabolic toxicity is not restricted to direct profiling of metabolites. Experimental and computational approaches investigating metabolism-related pathways, cellular homeostasis, and key molecular regulators can complement direct metabolite profiling and provide additional insights into how environmental chemicals perturb biological metabolism. Huang et al. (contribution 5) investigated the male reproductive toxicity of nano-antimony trioxide (Nano-Sb_2_O_3_). Exposure caused testicular Sb accumulation, impaired sperm quality, increased oxidative stress, and testicular pathological alterations. Transcriptomic analysis showed enrichment of the PPAR and PI3K–Akt signaling pathways and identified SPP1 as a prominently altered gene. Silencing Spp1 attenuated inflammatory responses and restored the expression of blood–testis barrier-associated proteins in Sertoli cells. These findings link internal nanomaterial exposure with metabolism-related signaling and reproductive toxicity. Wang et al. (contribution 6) investigated the role of PERK-mediated endoplasmic reticulum stress and autophagy in acrylamide-induced neurotoxicity. Acrylamide induced PERK pathway activation, apoptosis, and autophagy in SH-SY5Y cells, whereas inhibition experiments showed that PERK-mediated autophagy exerted an antiapoptotic effect. As autophagy is closely involved in intracellular substrate recycling and cellular metabolic homeostasis, this study provides additional insight into cellular metabolism-related responses to chemical exposure. Big-data-based computational toxicology is emerging as an efficient approach for linking chemical exposures with potential metabolic pathways and molecular targets. Lei et al. (contribution 7) applied network toxicology and molecular docking to investigate tetracycline-induced acute pancreatitis. The authors identified 259 potential exposure–disease targets and 22 core targets, including TP53, TNF, and AKT1. Functional enrichment implicated PI3K–Akt, MAPK, HIF-1, AGE–RAGE, and lipid metabolism-related pathways, while molecular docking was used to evaluate potential interactions between tetracycline and core targets. Jiang et al. (contribution 8) investigated the potential role of bifenthrin in recurrent implantation failure and recurrent pregnancy loss by integrating chemical target prediction, bulk and single-cell transcriptomic information, immune infiltration analysis, and molecular docking. BCL2, HMOX1, CYCS, and PTGS2 were identified as key genes, linking bifenthrin exposure with oxidative stress, mitochondrial function, lipid-related signaling, immune regulation, and reproductive outcomes. These studies illustrate how computational toxicology can utilize existing exposomic and biological information to prioritize potential molecular targets and metabolism-related pathways, providing hypotheses for subsequent metabolomic and experimental validation.

## 3. Conclusions

Further development of environmental chemical exposomics and metabolomics should focus on several interconnected directions. First, methodological development remains important for both exposomics and metabolomics. Emerging approaches such as single-cell metabolomics will enable metabolic responses to be characterized at the cellular level. Meanwhile, external and internal exposure assessment should be further integrated by continuously improving chemical exposome evaluation methods, developing rapid detection technologies, and incorporating personal and wearable-device-based approaches to better characterize individual exposures, strengthen the linkage between external and internal exposure, and identify major exposure sources. Second, the joint analysis of exposome and metabolome data should be strengthened through big data, artificial intelligence, and particularly computational toxicology to construct multidimensional “exposome–metabolome–disease” networks and identify potential molecular targets linking environmental exposures with health outcomes. Third, greater attention should be paid to metabolome remodeling as a central biological response connecting environmental exposures with disease. Beyond the conventional transcriptome–proteome–metabolome framework, emerging biological layers such as the gut microbiome and epigenome should be incorporated to clarify the multidimensional regulation of exposure-induced metabolic alterations. The eight articles in this Special Issue span external and internal exposure assessment, metabolomics, multi-omics integration, and experimental and computational investigations of metabolism-related mechanisms. Together, they enrich our understanding of environmental chemical exposomics and metabolomics in toxicology and provide new strategies and foundations for future research, health risk assessment, and disease prevention.

## Data Availability

No new data were created or analyzed in this study. Data sharing is not applicable to this article.
